# Genes, culture, and neural sensitivity to norm violations: a *DRD4* × culture interaction study

**DOI:** 10.1093/scan/nsaf083

**Published:** 2025-08-09

**Authors:** Cristina E Salvador, Kirby T Lam, Mayumi Karasawa, Anthony King, Nirmala Rajaram, Michele J Gelfand, Shinobu Kitayama

**Affiliations:** Department of Psychology and Neuroscience, Duke University, Box 90086, Durham, NC 27708, United States; Department of Psychology and Neuroscience, Duke University, Box 90086, Durham, NC 27708, United States; Department of Communication, Tokyo Woman’s Christian University, 2-chōme-6-1 Zenpukuji, Suginami City, Tokyo, 167-0041, Japan; Institute for Behavioral Medicine Research, Ohio State University, 460 Medical Center Drive, Columbus, OH 43210, United States; Department of Psychology and Department of Psychiatry, University of Michigan, 530 Church Street, Ann Arbor, MI 48109, United States; Department of Organizational Behavior, Stanford University, 655 Knight Way, Stanford, CA 94305, United States; Department of Psychology and Department of Psychiatry, University of Michigan, 530 Church Street, Ann Arbor, MI 48109, United States

**Keywords:** Culture, Social Norms, Gene x Culture, N400, EEG, DRD4

## Abstract

Cultures, such as Japan, are characterized by tighter or more rigid norms than others, like the United States. However, the mechanism underlying this cultural difference remains unclear. We tested the hypothesis that individuals carrying genetic polymorphisms linked to cultural learning, particularly the 7- or 2-repeat variable number of tandem repeat variants of the dopamine D4 receptor gene, *DRD4*, would show heightened sensitivity to norm violations if they are from tight cultures but not in loose cultures. A total of 214 Japanese and 236 European American young adults (total *N* = 450) evaluated the normativity of various behaviors while their electroencephalogram (EEG) was recorded. Consistent with previous findings, norm violations elicited a robust N400 response, an electrocortical marker of expectancy violation. Critically, this N400 norm-violation effect was significantly stronger for Japanese carriers of the *DRD4* alleles linked to cultural learning, but no such genetic moderation was observed among European Americans. Moreover, Japanese non-carriers showed a significantly weaker N400 response than their American counterparts. These results suggest that in a tight culture like Japan, heightened neural sensitivity to norm violations may be concentrated among individuals with genetic predispositions for enhanced reward processing, pointing to the dynamic interplay between genetic variations and cultural environments.

## Introduction

Social norms—shared rules to promote social coordination—are crucial for enabling humans to form complex social systems of meanings and practices, called culture (Gelfand et al. 2011, Henrich 2014, Kitayama and Salvador 2024). Cultures vary systematically in the strength of these norms and how strictly they are enforced, a dimension known as tightness-looseness (Gelfand et al. 2011). For instance, the United States exemplifies a loose culture, where people have greater freedom to deviate from social norms, whereas Japan is a tight culture where deviations are more likely to be attended to and punished. Previous research on tightness-looseness has largely relied on self-report and behavioral indices, leaving the underlying mechanisms poorly understood.

To address this gap, we take a neuroscience approach. First, we use a neural marker of spontaneous sensitivity to norm violations (Mu et al. 2015, Salvador et al. 2020a, Goto et al., 2022), allowing us to bypass self-presentation biases and related artifacts. Second, we examine the moderating role of the *DRD4*, previously linked to enhanced reward processing (Glazer et al. 2020) and greater susceptibility to environmental influences such as parenting quality (Belsky and Pluess 2009, van IJzendoorn et al. 2011). Recent work suggests that *DRD4* also modulates cultural learning (Kitayama et al. 2014, 2020, Yu et al. 2019). Drawing on this research, we hypothesized that Japanese individuals carrying *DRD4* variants linked to heightened reward sensitivity would also show increased neural sensitivity to norm violations—consistent with their tight cultural environment. In contrast, we expected no such genetic modulation among Americans, given the relative looseness of US culture.

### The role of *DRD4* in reward processing

*DRD4* is an intriguing candidate gene for examining cultural learning, due to its involvement in the mesolimbic and mesocortical dopamine systems involved in reward processing. *DRD4* genetic variants are also unique among numerous genes in that they are expressed in structurally and functionally distinct dopamine D4 proteins with differing affinities, intracellular signaling, and pharmacodynamic properties (Asghari et al. 1995, D’Souza et al. 2004). Of particular interest is a variable number of tandem repeat (VNTR) polymorphism located in exon 3 of the gene, consisting of a 48-base pair sequence repeated between 2 and 11 times. The most common variant is 4-repeat, while the 7- and 2-repeat variants account for a substantial minority. Other variants are relatively rare. Importantly, evidence suggests that the 7- and 2-repeat variants are linked to enhanced synaptic dopamine transmission, compared to the 4-repeat variant (Wang et al. 2004).

Dopamine plays a central role in reward processing regions of the brain, and the D4 receptor is also prevalent in the prefrontal cortical regions (Ferré et al. 2022). This suggests that reward sensitivity—the brain’s ability to detect external (including cultural) rewards and compute their contingencies with various behaviors—may be enhanced in individuals carrying the 7- or 2-repeat variant of *DRD4* (Glazer et al. 2020). Notably, these variants became more frequent during human migration out of Africa during the last 60 000 years, coinciding with the gradual development of culture as we know it today (Wang et al. 2004). Scholars have posited a role for this gene in human migration since the population-level prevalence of these gene variants increases with migratory distance from Africa (Chen et al. 1999, Matthews and Butler 2011). This literature suggests that *DRD4* may have co-evolved with culture by enhancing the capability to compute cultural reward contingencies, which in turn may have facilitated the development of increasingly complex and elaborate social norms (Kitayama et al. 2016, Kitayama and Yu 2020, Kitayama and Salvador 2024). One hypothesis is that the 7- and 2-repeat variants of *DRD4*, incorporated into the human genome relatively recently in evolutionary terms, have increased the efficiency of reinforcement learning (Kitayama et al. 2016), promoting behavioral traits consistently reinforced in a given culture, such as independence in Western societies and interdependence and East Asian societies.

### Interactions between *DRD4* and culture

The putative co-evolution of the 7- or 2-repeat variant of *DRD4* and culture suggests that carriers of these variants may differ significantly from noncarriers on various cultural dimensions. Supporting this possibility, an early self-report-based study found that East Asians are relatively more interdependent (valuing social harmony) and less independent (valuing autonomy less) compared to European Americans (Kitayama et al. 2014). Importantly, this cultural difference was observed among the 7- or 2-repeat *DRD4* variant carriers but not among non-carriers. While self-report studies are an important first step, they are detached from the direct operation of genes. To build a stronger bridge between genes and behavior, it is crucial to test endophenotypes—neural processes likely serving as intermediate steps in the causal sequence (Kendler and Neale 2010).

Subsequent studies have addressed this gap. If independent behaviors, such as goal-pursuit and strategizing, require both value-based decision making and abstract self-concepts, one might expect that engagement with independent cultures would entail increased use of the prefrontal regions. These regions include the orbitofrontal cortex (OFC), involved in value-based decision making (Fellows 2011, O’Doherty 2011), and the medial prefrontal cortex (mPFC), involved in abstract ideation about the self (Northoff 2010, Ma et al. 2014). Frequent engagement of these regions may, through neuroplasticity, result in increased gray matter volume, as shown in previous studies on cab driving (Maguire et al. 2000), musical training (Olszewska et al. 2021), and other cognitively demanding tasks (Scholz et al. 2009).

Consistent with this literature, research shows that the prefrontal regions, including OFC and mPFC, are significantly larger in volume for European Americans and East Asians, after controlling for age, sex, and total brain volume. Notably, this cultural difference is observed only among the carriers of the 7- or 2-repeat variants of *DRD4* (Yu et al. 2019). Different regions are larger amongst East Asians than European Americans. Assuming that interdependent behaviors require specific interpersonal cognitive operations, such as perspective taking and mind reading, we may expect greater engagement of the temporo-parietal junction (TPJ), a region of the brain uniquely linked to these cognitive operations (Martin et al. 2020). Indeed, TPJ volume tends to be greater for East Asians compared to European Americans, but again only among carriers of the 7- or 2-repeat variant of *DRD4* (Kitayama et al. 2020).

### *DRD4*, norm violation detection, and cultural tightness-looseness

So far, the genetic modulation of cultural acquisition has yet to be tested on sensitivity to norm violations. In tight cultures (e.g. Japan), norm compliance is strongly and consistently rewarded, while deviances are punished (Gelfand et al. 2011). This cultural pressure may foster the development of neural mechanisms specialized for the rapid detection of norm violations. These mechanisms are likely grounded in dopamine-based reward systems. Detecting norm violations may be inherently rewarding in tight cultural contexts, and the associated neural reinforcement may further enhance the sensitivity to these violations over time. This positive feedback loop may be especially pronounced in individuals with genetic variants associated with heightened reward sensitivity—such as carriers of the 7- or 2-repeat alleles of the *DRD4* gene—mirroring previously reported *DRD4 *× culture interactions.

What about loose cultures, such as the United States? In these contexts, norm compliance is not consistently rewarded, nor are norm violations reliably punished. Although individuals in such cultures do learn which behaviors conform to prevailing norms, they are unlikely to be systematically rewarded for detecting norm violations. As a result, the development of dopamine-based mechanisms for norm violation detection may be less robust in these cultures, regardless of individuals’ genetic sensitivity to rewards. Consequently, we may expect relatively low sensitivity to norm violations across *DRD4* variants in these cultures.

According to this analysis, Japanese carriers of the 7- or 2-repeat alleles should be more sensitive to norm violations than Japanese non-carriers. Moreover, like Japanese non-carriers, European Americans are expected to show relatively low sensitivity to norm violations regardless of carrier status. Since the *DRD4* alleles of interest are less frequent than the common 4-repeat variant, research that does not account for *DRD4* polymorphic variation may fail to detect robust cultural differences in norm sensitivity.

### Present research

Building on earlier studies (Mu et al. 2015, Goto et al. 2022), we utilized an Event Related Potential (ERP) marker, called N400, known to signal the detection of semantic inconsistencies, including norm violations. Testing a large sample of both European Americans in the United States (*N* = 197) and Japanese in Japan (*N* = 178) (total *N* = 375), we anticipated an increase in N400 in response to norm-violating behaviors (e.g. singing in the hospital) compared to normal behaviors (e.g. singing in the choir performance) regardless of Culture. Notably, one prior study by Mu et al. (2015) found a significant cultural difference in the frontal and temporal parietal regions, with the norm-violation N400 being significantly greater for East Asians than European Americans. Another study, however, failed to find a similar cultural difference (Goto et al. 2022). Significantly, neither study tested the possible moderating role of *DRD4*. Here, we employed a large sample size and tested the hypothesis that the predicted cultural difference, with East Asians showing a stronger norm-violation N400 than European Americans, would be observed primarily among carriers of the 7- or 2-repeat variant of *DRD4*. Additionally, we explored whether this cultural difference might be more pronounced in the frontal regions compared to other brain areas.

## Methods

### Sample

We prescreened participants at each site to be of the ethnicities of interest (European American in the USA and Japanese in Japan) and eligible for EEG studies. We confirmed their eligibility through demographic questionnaires during the study. Two hundred thirty-six European American undergraduates at the University of Michigan (66 males and 166 females, with 4 undisclosed) and 214 Japanese undergraduates at several universities in the broad Tokyo area (69 males and 122 females, with 23 undisclosed) participated in the study. A past cross-cultural study by Mu et al. (2015) with the same EEG measure used *N* = 25 per culture. However, they did not examine the effect of genes. In contrast, Yu et al. (2019) examined the effect of genes on regional brain volume, with a target sample of 26–37 per cell defined by culture and *DRD4* genotype, *N* = 63 per culture. To guard against false positives, we tripled the sample size. Further, this will enable us to gain a sufficient number of carriers among the Japanese since the percentage of carriers of *DRD4* 7R/2R variants is low in general populations (combined global mean = 28.8%) (Chang et al. 1996).

European American participants were tested in an EEG lab at the University of Michigan (Ann Arbor, Michigan), whereas Japanese participants were tested in a comparable EEG lab at Tokyo Woman’s Christian University (Suginami, Tokyo). They were compensated $80 or its rough equivalent in Japanese currency (10 000 yen) for their participation. In the USA, they were compensated $30 for their participation. Due to a history of medication, prior head injury, and excessive noise in EEG data (Luck, 2014), 39 European American and 36 Japanese participants were excluded from the analysis. Out of 197 American participants who had usable EEG data, 47% (*n* = 94) carried the 7/2-repeat allele. Out of 178 Japanese participants with usable data, only 15% (*n* = 28) carried this allele. The American percentage is consistent with prior evidence previously observed for European American samples (Sasaki et al. 2013, Kitayama et al. 2014). The Japanese percentage was lower; yet even the smallest cell of Japanese carriers (*N* = 28) had a sample size comparable to each of the two cultural groups tested in Mu et al. (2015) (*N* = 25) and Goto et al. (2022) (*N *= 27–32).

### Procedure

#### Norm violation judgment task

**Figure 1.**
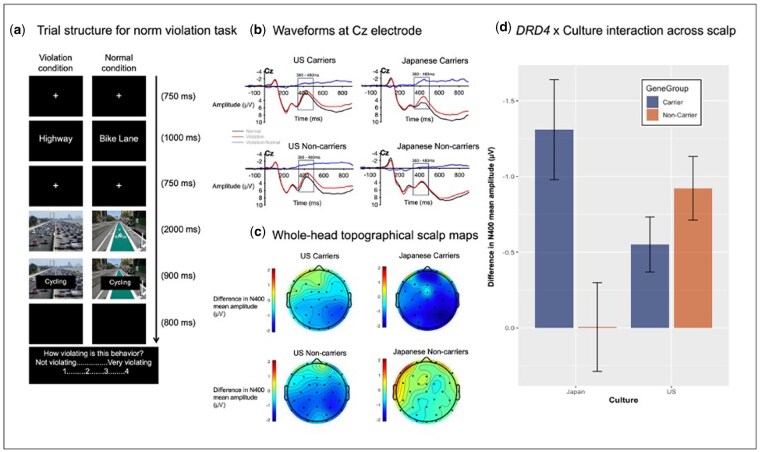
The DRD4 × culture interaction in norm violation detection as captured by increased N400 responses to norm-violating (vs. normal) behaviors. (a) Trial structure showing the sequence of stimulus presentation, participant responses, and their respective timing. (b) EEG waveforms at the electrode Cz for the four conditions defined by *DRD4* genotypes and cultural background. (c) Scalp maps depicting the spatial distribution of the N400 response to norm-violating versus normal behaviors, with larger effects shown in darker shades of blue, in each of the four conditions. (d) The magnitude of N400 response differences between norm-violating and normal behaviors, quantified by DRD4 genotype and cultural background.

This task involved a series of judgments on how severely norm-violating or normal various behaviors are in different settings. As shown in Fig. 1a, each trial started with a fixation point (‘+’) presented for 750 ms at the center of the computer screen. Then, a word representing a location or situation (e.g. bike lane) was presented for 1000 ms, followed by another fixation point for 750 ms, after which a picture of that location or situation was shown. 2000 ms afterward, a word representing a behavior (e.g. cycling) was superimposed on the picture for 900 ms. Participants were instructed to imagine someone performing the behavior in the location or situation (e.g. ‘cycling on the bike lane’). The situation-behavior pairs were adopted from two studies (Salvador et al. 2020a, 2020b). Then, a prompt appeared on the screen, upon which participants reported how violating the behavior would be in the situation by choosing a number from a 4-point rating scale ranging from 1 (= normal) to 4 (= very violating). Before the response prompt, there was an 800 ms interval to ensure that the response would not interfere with ERPs evoked by the behavior. There were 102 unique trials, which were repeated once to yield 204 trials. The trials were presented in two blocks and randomized for each participant.

The European American participants received materials in English and with pictures from North American contexts. Japanese participants received materials in Japanese and included scenes from Japan. We translated these English stimuli into Japanese with a translation-to-back-translation procedure with fluent Japanese-English bilingual speakers.

#### EEG recording and processing

Participants were individually tested in an EEG lab. Upon arrival at the lab, participants filled out a consent form and prescreening questions on medication use, history of seizure disorders, head injury, ethnicity, and handedness. Participants were then seated ∼60 cm from a color computer display. After the EEG was set up, participants performed the norm violation judgment task wearing the EEG cap. EEG was recorded with 32-channel electrodes using the BioSemi ActiveTwo System. Six external electrodes were used for ocular correction. The data were digitized at the rate of 512 Hz and resampled at 256 Hz, and then offline re-referenced to the average of the two mastoids. The data were analyzed using MATLAB with EEGLAB plugin and ERPLAB extension. An offline butterworth filter with a lowpass of 20 Hz and a high pass of 0.1 Hz was applied. Then the data were segmented into a 200 ms prestimulus baseline and 800 ms post-feedback (1000 ms in total), and baseline-corrected before the presentation of the stimulus. Before artifact detection, the data were visually inspected for bad electrodes, which were subsequently interpolated using spherical interpolation. Trials were rejected if they exceeded ± 100 mv as determined with a 200 ms moving window with a 50 ms step threshold, if they fluctuated >30 mv between two sampling points, or if they had little to no activity (under .5 mv) over the course of the trial. Trials with blinks occurring ±100 ms around the onset of the stimulus behavior were removed to ensure that the behavior was appropriately attended. Participants with <50% of usable trials were excluded from the analysis. On average, participants had 87.92% of trials available. All other trials containing blink ocular artifacts were corrected based on a commonly used algorithm (Gratton et al. 1983).

To extract the N400, we first baseline-corrected the data to 200 ms prior to the critical stimulus. As in prior work, we observed a negative-going deflection ∼430 ms after the onset of the behavior in the central sites (Fig. 1b). We extracted the mean amplitude using a time window ±50 ms around the average peak latency (380–480 ms). Due to the inconsistency in localization of the N400 effect across previous studies (Mu et al. 2015, Salvador et al. 2020a, Goto et al., 2022), we calculated a whole-head average for the mean in each of the three stimuli conditions (Normal, Weak violation, Strong violation). In repeated-measures analysis of variance (ANOVA)s on the difference in the whole-head N400 across conditions, there was no significant difference between the weak and strong violation conditions, *P* = .633. Thus, the main dependent variable was a difference score by subtracting each participant’s normal condition mean amplitude from the average of their weak and strong violation condition amplitudes like prior work by Salvador et al. (2020). When a repeated-measures ANOVA with all three conditions was used as a dependent variable (DV) instead, the results are similar (see Table S2). Since the N400 appeared to be present across these sites (Fig. 1c), we first analyzed the whole-head average N400 in a Culture × *DRD4* ANOVA as the main analysis. We also conducted Culture × *DRD4* ANOVAs on the individual Fz, Cz, Pz, and T8 electrode sites (see Table S3 and Fig. S1) to compare with prior work (Mu et al. 2015, Salvador et al. 2020a, 2020b, Kitayama et al. 2023).

#### *DRD4* assays

To assess the *DRD4* 7R/2R status, we had participants use an Oragene saliva kit (OG-500) (DNA Genotek, Kanata, Ontario, Canada). Genomic DNA was extracted using a high-capacity membrane-based column (QuickGene810, AutoGen, Inc., Holliston, MA) and was quantitated using an A260/A280 ratio with a NanoDrop spectrophotometer (Thermo Fisher Scientific, Inc., Wilmington, DE) and agarose gel electrophoresis. The *DRD4* VNTR polymorphism was amplified with 0.2 µM of *DRD4* forward primer 5′-GCGACTACGTGGTCTACTCG and 0.2 µM of *DRD4* reverse primer 5′-AGGACCCTCATGGCCTTG (Lichter et al. 1993) using the Roche GC-Rich PCR System amplification buffer (Roche Applied Science, Inc., Mannheim, Germany) and 20 ng of genomic DNA in a volume of 25 µl. The samples were heated in a LightCycler^®^ 96 Instrument (Roche Diagnostics Corporation, Indianapolis, IN) at 95°C for 3 min, then cycled 40 times at 95°C for 20 s, 57°C for 20 s, and 72°C for 1 min, followed by 72°C for 3 min. Polymerase chain reaction products were separated and visualized on a 2% agarose gel (type 1-A, Sigma, St Louis, MO) stained with ethidium bromide.

#### Cultural dimensions

Participants filled out a packet of questionnaires before they were dismissed. The packet included a modified version of the Singelis self-construal (SC) scale (Park and Kitayama 2014). The scale was composed of a 10-item Independent SC subscale (e.g. ‘I do my own thing regardless of what others think’) (α = 0.77 in Japan, α = 0.74 in the United States) and a 10-item Interdependent SC subscale (e.g. ‘I will sacrifice my self-interest for the benefit of the group I am in.’) (α = 0.71 in Japan, α = 0.64 in the United States). These judgments were made on a 5-point rating scale from 1 (Doesn’t describe me at all) to 5 (Describes me very much). We collapsed these two subscales into an aggregate SC measure where positive values indicated more independent SC and negative values for interdependent orientations. They also filled out a norm tightness/looseness scale (Gelfand et al. 2011) (α = 0.75 in Japan, α = 0.81 in the United States). The scale was composed of 14 items (e.g. ‘In this country, there are very clear expectations for how people should act in most situations’), which they judged their level of agreement on a 1–6 scale. For exploratory analyses, participants filled out several additional questionnaires.

### Statistical analysis

For the current analyses, we conducted a 2 (Culture: USA, Japan) × 2 (*DRD4* status: carrier, non-carrier) between-subjects ANOVA. Our main dependent variable was the difference in N400 mean amplitudes between violating and normal behaviors for the whole head average. The post-processed data cleaning and analyses were conducted on RStudio and Jamovi.

## Results

### Self-report measures: 1. Perceived tightness vs. looseness of social norms and SC

We employed the Park and Kitayama (2014) version of the Singelis SC scale to assess independent (vs. interdependent) SC. Consistent with past work, European Americans rated themselves more independent, or less interdependent (*M *= 0.19, *SD *= 1.14) than Japanese (*M *=* −*0.52, *SD *= 1.23), *t*(354) = 5.61, *P* < .001, Cohen’s *d *= 0.60. However, unlike previous work (Kitayama et al. 2014), these cultural differences were observed equally for both carriers and non-carriers of the 7- or 2-repeat allele of *DRD4* (Table S1), *F*(1,352) = 0.06, *P* = .808.

We used the Gelfand et al. (2011) scale to assess the tightness vs. looseness of social norms. As predicted, European Americans rated their norms looser (or less tight) (*M *= 3.63, *SD *= 0.62) than Japanese (*M *= 4.37, *SD *= 0.50), *t*(353) = −12.20, *P* < .001, Cohen’s *d *=* −*1.30. The main effect of *DRD4* status was not significant, *F*(1, 351) = .70, *P* = .40, np2 = .002. The *DRD4* × Culture interaction on the TL scale did not reach statistical significance, *F*(1, 351) = 3.32, *P* = .069, np2 = .009 (A subsequent simple effects test revealed that European American carriers reported being looser (*M* = 3.53, *SE* = .059) than European American non-carriers (*M* = 3.72, *SE* = .056), *t*(351) = −2.39, *P* = .018. In contrast, Japanese carriers (*M* = 4.43, *SE* = .11) and non-carriers (*M* = 4.36, *SE* = .049) did not differ from each other, *t*(351) = .59, *P* = .55. Given the marginal nature of the *DRD4* × Culture interaction, it is unclear whether this pattern is robust.).

### Self-report measures: 2. Perceived norm violations

Participants were presented with 102 behaviors that are presented in either congruous contexts (rendering them ‘normal’) or moderately or extremely incongruous contexts (rendering them moderately or extremely ‘norm-violating’) (see Fig. 1a). There was a significant main effect of Condition, *F*(2, 734) = 8073.52, *P* < .001, *η*^2^ = 0.747. Both European Americans and Japanese rated extremely violating behaviors as strongly violating (*M *= 3.33, *SD *= 0.40), followed by moderately violating behaviors (*M *= 2.51, *SD *= 0.37), with normal behaviors as least violating (*M *= 1.13, *SD *= 0.10). Japanese ratings of norm violating stimuli (Strong: *M *= 3.50, *SD *= 0.42; Moderate: *M *= 2.60, *SD *= 0.38) were significantly greater than European Americans ratings of norm-violating (but not normal) behaviors (Strong: *M *= 3.17, *SD *= 0.30; Moderate: *M *= 2.42, *SD *= 0.34), resulting in a significant Culture main effect, *F*(1,367) = 35.83, *P* < .001, *η*^2^ = 0.007, and Culture × Condition interaction, *F*(2, 734) = 35.53, *P* < .001, *η*^2^ = 0.003. This pattern was not moderated by *DRD4*, as shown by Table 1, with no significant interactions involving *DRD4*, *F*(2, 734) = 1.98, *P* = .139, *η*^2^ = 0.000.

**Table 1.** Means and standard deviations of stimuli ratings across conditions, cultures, and DRD4 variant carrier status.

Condition	Japanese		American	
	Carriers	Non-carriers	Carriers	Non-carriers
Strongly violating	3.48 (0.38)	3.51 (0.43)	3.19 (0.31)	3.15 (0.29)
Moderately violating	2.51 (0.45)	2.62 (0.37)	2.46 (0.36)	2.39 (0.33)
Normal	1.10 (0.13)	1.16 (0.12)	1.09 (0.09)	1.09 (0.07)

### ERP measures

The relevant mean amplitudes of the norm-violation N400 are plotted in Fig. 1d. Of note, neither Culture nor *DRD4* main effects reached statistical significance, *F*(1, 371) = 0.06, *P* = .814, *η*^2^ = 0.000 and *F*(1, 371) = 1.85, *P* = .175, *η*^2^ = 0.005. Importantly, the Culture × *DRD4* interaction proved significant, *F*(1, 371) = 5.98, *P* = .015, *η*^2^ = 0.016. The norm-violation N400 was significantly greater for the 7- or 2-repeat carriers (*M *=* −*1.31, *SD *= 1.74) than for the non-carriers (*M *=* −*0.01, *SD *= 3.59) among Japanese, *t*(371) = *−*2.31, *P* = .021. The corresponding difference was negligible among European Americans between carriers (*M *=* −*0.55, *SD *= 1.77) and non-carriers (*M *=* −*0.92, *SD *= 2.13), *t*(371) = 0.95, *P* = .342. Among carriers, Japanese exhibited a somewhat greater N400 than their European American counterparts, but the difference did not reach statistical significance, *t*(371) = *−*1.29, *P* = .200. Among non-carriers, Japanese displayed a significantly less norm-violation N400 than their European American counterparts, *t*(371) = 2.62, *P* = .009. We also tested specific electrodes, particularly, Fz, Cz, Pz, and T8 and found very similar patterns (Fig. S1).

We subsequently tested correlations between the norm-violation N400 and the self-report measures, which included perceived tightness (vs. looseness) of cultural norms, SC, and perceived violation of norm-violating (vs. normal) behaviors. As shown in Table 2, the only significant correlation was a positive relationship between perceived norm tightness and the ratings of how violating norm violating versus normal behaviors were rated.

**Table 2.** Correlations between self-construal, norm tightness/looseness, stimuli ratings, and norm-violating N400 among European American and Japanese participants. Significance is denoted with asterisks based on the p-values.

	Japanese				European/White American			
Variable	SCS	TLS	Rating diff.	N400 diff.	SCS	TLS	Rating diff.	N400 diff.
1. Self-construal (SCS)	–	−.054	−.032	−.022	–	−.109	.043	−.065
2. Tight/loose (TLS)		–	.265***	.060		–	.144*	−.033
3. Rating difference			–	−.124			–	.012
4. N400 difference				–				–

* *P* < .05.

*** *P* < .001.

## Discussion

### The crucial role of *DRD4*

While most aspects of culture, including cultural norms, are learned through socialization (Greenfield et al. 2003), the learning of culture is genetically modulated (Kitayama et al. 2016, Kitayama and Salvador 2024). This proposition has received support in a series of recent studies documenting the modulation of cultural differences by variable number of tandem repeat (VNTR) variants of the *DRD4* (Yu et al. 2019, Glazer et al. 2020, Kitayama et al. 2020, Kitayama and Yu 2020). Our current work built on this literature and explored the impact of the *DRD4* VNTR variants on neural response to norm violations and provided new insights.

In the analysis of N400 responses to norm-violating behaviors compared to normal behaviors, no significant main effect of Culture was observed. We found no robust cultural difference in the norm-violation N400 when disregarding *DRD4*—a critical regulator of cultural learning. This finding is consistent with a recent study by Goto et al. (2022), which failed to replicate Mu et al. (2015). However, unlike these previous studies, we examined *DRD4* variants of our participants and found a significant interaction between *DRD4* and Culture on norm-violation N400.

The resulting pattern was intriguing. Among Japanese participants, those carrying the 7- or 2-repeat variant of the *DRD4* gene showed a stronger N400 response to norm violations compared to noncarriers. This pattern was not observed among European Americans, where carriers and noncarriers responded similarly. When comparing the two cultural groups, Japanese noncarriers showed a significantly weaker norm-violation response than European American noncarriers, whereas Japanese carriers showed a non-significant trend toward a stronger response than their European American counterparts. Since carriers and noncarriers exhibited cultural differences in opposite directions, there was no overall main effect of culture. This pattern replicates the findings of Goto et al. (2022), but our work adds an important nuance by demonstrating a Culture × *DRD4* interaction.

### Social dynamic of tight societies

Two specific observations suggest an intriguing social dynamic in tight societies. First, Japanese carriers of the *DRD4* 7- or 2-repeat variants exhibited significantly greater norm-violation N400 responses than non-carriers. This finding is consistent with the hypothesis that Japanese culture, known for its tight social norms, emphasizes detecting and punishing norm violations. Accordingly, individuals may be rewarded not only for conforming to social norms, but also for carefully monitoring and thus ‘policing’ others’ violations of these norms. Hence, the ability to effectively detect norm-violation is consistently rewarded, especially for the carriers of these *DRD4* variants, who are predisposed toward heightened reward processing. Moreover, this finding is in line with a well-known finding that East Asians exhibit holistic cognition (Nisbett et al. 2001, Nisbett 2010). Such cognition may facilitate the monitoring of others’ norm-violating behaviors, further reinforcing its value in tight social environments.

Second, carriers formed a small minority in the Japanese sample, comprising only 15% (*n* = 28 out of the total of 178 usable participants). One potential concern is that this sample is too small. However, this sample size is comparable and slightly larger than the samples used in past work examining cultural variation with the same N400 index (Mu et al. 2015, Goto et al. 2022). The remaining Japanese participants, carrying neither the 7- nor the 2-repeat allele, showed very low levels of norm sensitivity—even lower than their American counterparts. How can this finding be reconciled with the premise that Japan is a tight society? We suggest that, in societies with strong social norms, a relatively small group of individuals, highly attuned to norm violations, guards and enforces these norms. Once violations are detected, these individuals attempt to enforce the norms—a behavior that is socially approved precisely because norms are tight. Thus, tight societies are regulated primarily at the group level. As a result, the remaining individuals may not need to attend closely to norm violations themselves, relying instead on the subgroup specializing in norm enforcement.

Although direct evidence for this division of labor is current lacking, our analysis is consistent with a recent theoretical proposal: only a relatively small proportion of people may internalize their cultural values and beliefs [called believers (The Hernandez et al. (2022) paper refers to this group as well-socialized members of society. We adopt the term believers instead since it is brief and has a similar meaning.)], while the rest (called opportunists) pursue self-interest within the system established by the believers (Hernandez et al. 2022). Paradoxically, although the opportunists do not believe in the cultures’ contingencies, they end up reinforcing the culture they exploit, because their actions are shaped by the cultural payoff structure. In our case, the carriers function as the believers, attuned and committed to norm enforcement, while non-carriers resemble opportunists. Although non-carriers may not be sensitive to norms or care about them, they nonetheless help sustain Japan’s tightness simply by navigating the cultural system with knowledge of the norms. Yet, because they are not believers, they do not show increased neural sensitivity to norm violations. This dynamic leaves the small minority of carriers as the primary ‘guardians’ of social norms.

This analysis also clarifies another otherwise puzzling result: While Japanese carriers showed a somewhat stronger norm-violation N400 than European Americans, this cultural difference did not reach statistical significance. Instead, Japanese non-carriers showed a significantly *less* pronounced norm-violation N400 than their European American counterparts. This supports the idea that the tight norms of Japanese society are regulated primarily by a small minority of individuals (the carriers in our case) specializing in norm enforcement.

### Absence of *DRD4* effect in loose societies

It is notable that, unlike Japanese participants who showed a clear *DRD4* effect, European American carriers and non-carriers did not differ from each other. At first glance, this might seem puzzling. If the 7- and 2-repeat alleles of the *DRD4* gene amplify cultural influences, then one might expect the American tendency toward norm looseness—manifested as lower sensitivity to norm violations—to be even more pronounced among carriers of these variants. However, this apparent puzzle dissolves when we consider the underlying mechanisms more closely. In tight cultures such as Japan, detecting norm violations is likely to be consistently rewarded, especially for individuals with heightened genetic sensitivity to rewards, leading to greater efficiency in norm violation detection. In contrast, in loose cultures like the USA, such detection is not systematically reinforced. As a result, European Americans tend to be less sensitive to norm violations overall, and this effect is not moderated by genetic variations in reward sensitivity. Thus, the absence of a *DRD4* effect in the USA is consistent with the lack of culturally reinforced reward mechanisms for norm detection.

In short, in loose societies, individuals are aware of social norms. They therefore take note of norm violations when others’ behaviors do not comply with the norms, as revealed in the norm violation N400 effect. However, this detection of norm violations is not consistently rewarded—a feature of loose societies. Consequently, the norm violation N400 effect is not moderated by *DRD4*.

### Neural vs. self-report measures

In contrast to the N400 measure, which showed a significant *DRD4 ×* Culture interaction, self-report ratings of norm violation severity did not show this pattern. One possibility is that the effect of *DRD4* might be more likely to emerge in neural measures than in self-reports. We hypothesize that *DRD4* regulates reward sensitivity, which in turn modulates brain mechanisms involved in detecting norm violations. This spontaneous detection might eventually influence downstream responses, including self-reported severity judgments. However, the impact of *DRD4* may be expected to be more robust in the neural measure (often called endophenotype, Kendler and Neale 2010), which taps the brain mechanisms influenced directly by *DRD4*, than in the self-report measure, which is downstream. Indeed, much of the evidence on *DRD4* × Culture interactions is utilized neural indices (Yu et al. 2019, Glazer et al. 2020, Kitayama et al. 2020). In contrast, the original self-report finding (Kitayama et al. 2014) may not be as robust (Ishii et al. 2021).

Another possibility is that *DRD4* influences the spontaneous detection of norm violations but not the later appraisal of them. This interpretation is consistent with the idea that in tight societies, individuals who rapidly detect norm violations are consistently rewarded, producing a systematic *DRD4* effect. In contrast, appraising behaviors as norm-violating may not be socially rewarded unless it occurs quickly. This could explain why *DRD4* effects emerge in neural measures of detection but not in self-reported appraisals. Future work is needed to test both detection and appraisal processes using neural and self-report methods.

### Limitations and conclusion

While our study provides novel insights into the interaction between genetic predispositions, cultural norms, and neural responses to norm violations, several limitations should be acknowledged. First, although sufficiently large, our sample consisted of college students from Japan and the United States, which may limit the generalizability of our findings to other populations or age groups. Second, while we focused on the *DRD4* gene as a potential modulator of neural responses to norm violations, it is essential to recognize that genetic influences are complex and multifaceted. Other genetic polymorphisms and gene-environment interactions may also play significant roles in shaping individual differences in cultural sensitivity and norm enforcement. Moving forward, future research could explore additional genetic markers and environmental factors that may contribute to individual differences in cultural sensitivity and norm enforcement. Longitudinal studies could also investigate how these factors interact and evolve over time, shedding light on the dynamic nature of cultural learning and its neural correlates. Last but not least, the time is ripe to go beyond East and West, extending all work on culture including the current one to other regions of the globe (Kitayama et al. 2022, Kitayama and Salvador 2024).

In conclusion, our study contributes to the growing body of research on the interaction between genetics, culture, and neural processes. By elucidating the mechanisms underlying cultural learning and norm enforcement, we can deepen our understanding of human social behavior. This effort may inform interventions aimed at promoting cultural understanding and cooperation in an increasingly diverse world.

## Supplementary Material

nsaf083_Supplementary_Data

## Data Availability

The data underlying this article are available on Open Science Framework (OSF) at: https://osf.io/p8hav/? view_only=4b70abd4b21a42cd87c0e43815b2201d.
