# Supplementary material for: Genes, culture, and neural sensitivity to norm violations: a DRD4 × culture interaction study
Source: Soc Cogn Affect Neurosci. 2025 Aug 9;20(1):nsaf083. doi: 10.1093/scan/nsaf083 (PMC12422781; doi:10.1093/scan/nsaf083)
Supplement: nsaf083_Supplementary_Data [file nsaf083_supplementary_data.zip › scan-25-028-32-35.pdf]

Supporting Information for

Genes, Culture, and Neural Sensitivity to Norm Violations:

A *DRD4* x Culture Interaction Study

Cristina E. Salvador[1]

Kirby Lam[1]

Mayumi Karasawa[2]

Anthony King[3]

Nirmala Rajaram[4]

Michele J. Gelfand[5]

Shinobu Kitayama[4]

Key words: Culture, Social Norms, DRD4, Gene x Culture Interaction, EEG, N400

[1]Duke University, [2]Tokyo Women's Christian University, [3]Ohio State University,

[4]University of Michigan, [5]Stanford University

For Peer Review

Supplementary Tables & Figures

Table S1.
Self-Construal Differences between DRD4 Variant Carriers

Variable	*DRD4* variant carrier		*DRD4* variant non-carrier		*t*	*df*	*p*
	M	*SD*	*M*	*SD*			
Self-construal	0.00	1.17	-0.20	1.26	1.42	354	0.158

Note. Higher values on the self-construal scale indicate greater independent self-orientations.

Table S2.
Repeated Measures ANOVA between Culture, DRD4 Status, and Condition Type across the Whole Lobe

Within Subject Effects	*df*	*MS*	*F*	*p*	η^2
Condition Type	2	42.30	9.22	< .001	.001
Condition x Culture	2	7.54	1.64	.194	.000
Condition x *DRD4*	2	5.13	1.12	.327	.000
Condition x Culture x *DRD4*	2	15.00	3.27	.039	.001
Residual Error	742	4.59			

Between Subject Effects	*df*	*MS*	*F*	*p*	η^2
Culture	1	210.88	4.38	.037	.004
DRD4	1	0.62	0.01	.910	.000
Culture x *DRD4*	1	6.28	0.13	.718	.000
Residual Error	371	48.15			

Note. Culture has two levels: Japanese and European Americans. *DRD4* Status has two levels: carrier and non-carrier. Condition Type has three norm conditions: normal, moderately norm-violating, and extremely norm-violating behavior.

Table S3.
Between-subjects ANOVA between Culture and DRD4 Status for the Norm-Violation N400 at Temporal Lobe

	df	*MS*	*F*	*p*	η^2
Culture	1	0.28	0.04	.835	.000
DRD4	1	26.57	4.15	.042	.011
Culture x *DRD4*	1	60.99	9.53	.002	.025
Residual Error	371	6.40			

Note. Channel T7 was not recorded for Japanese participants, so we only present results from the T8 channel. Culture has two levels: Japanese and European Americans. *DRD4* Status has two levels: carrier and non-carrier.

Fig. S1.
The Culture x DRD4 interaction for the N400 difference between the average of the violation conditions and normal behavior condition at the electrode sites Fz, Cz, Pz, and T8.

For Peer Review

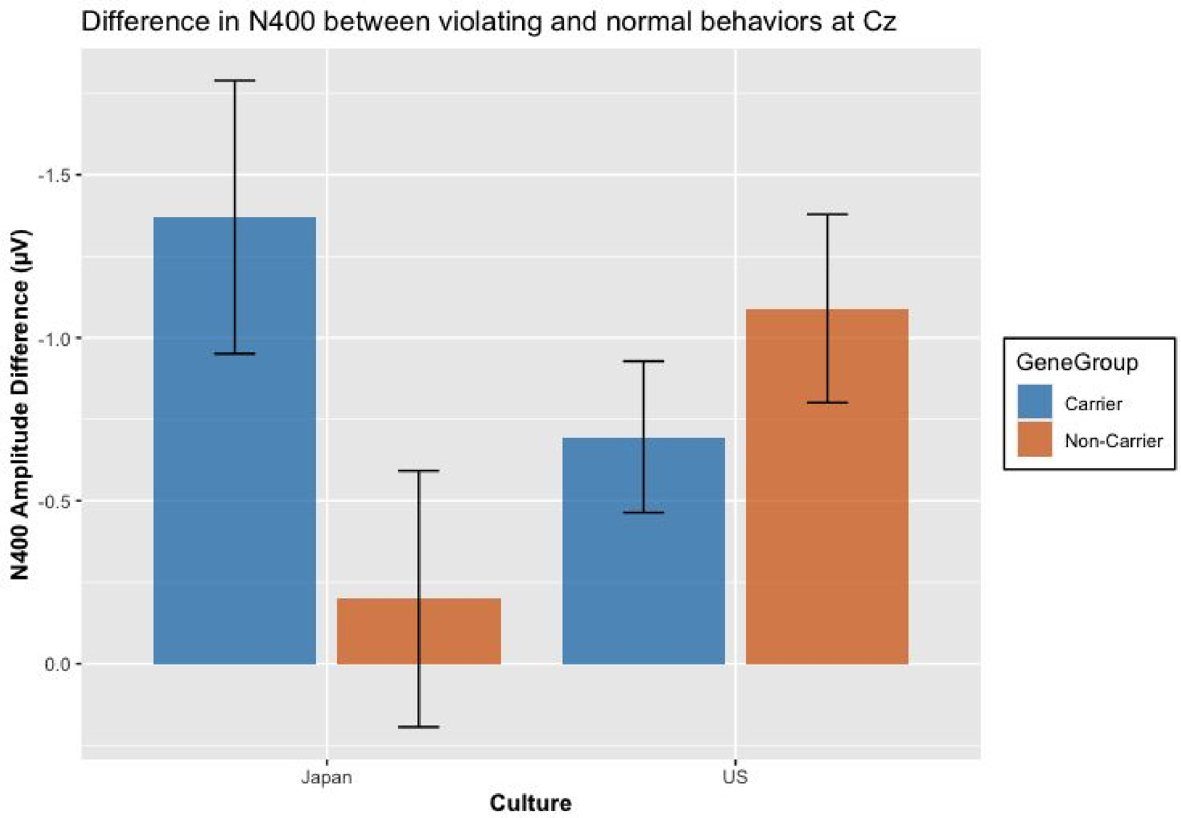
Difference in N400 between violating and normal behaviors at Cz
N400 Amplitude Difference (μV)
-1.5
-1.0
-0.5
0.0
Japan
US
Culture
GeneGroup
Carrier
Non-Carrier

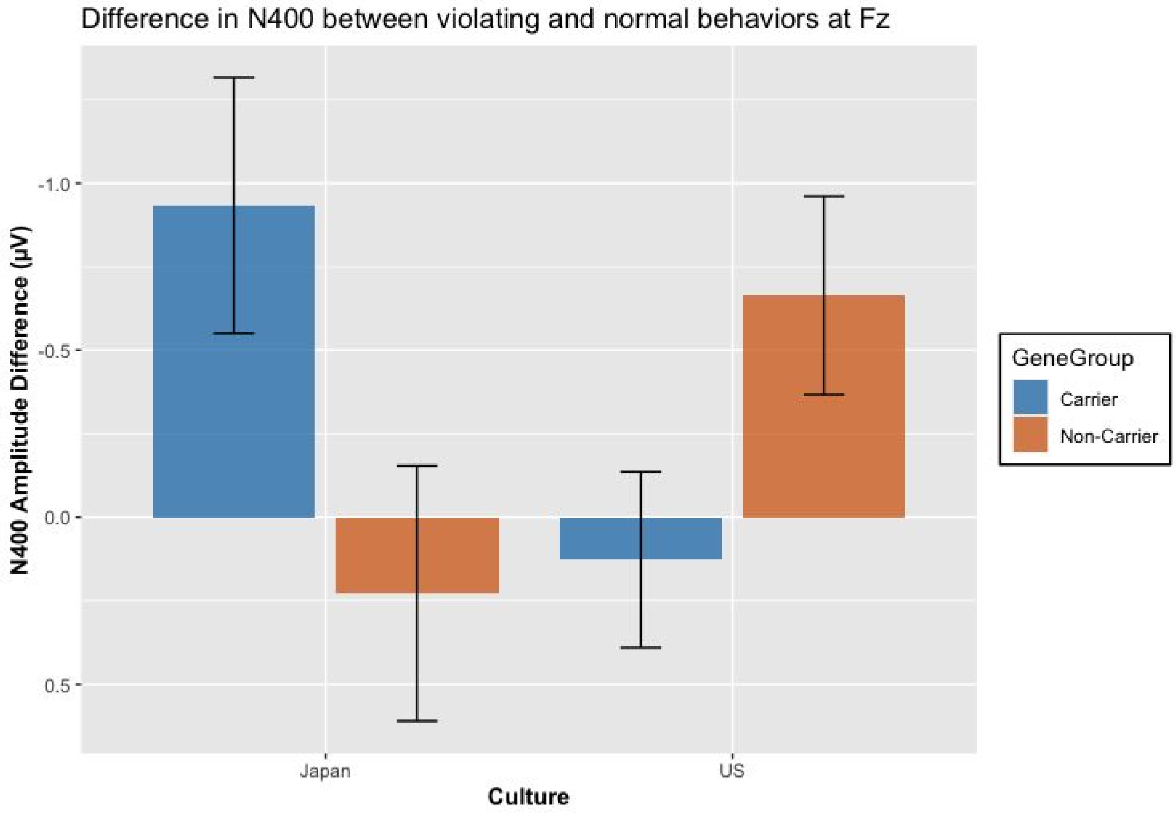
Difference in N400 between violating and normal behaviors at Fz
N400 Amplitude Difference (μV)
-1.0
-0.5
0.0
0.5
Japan
US
Culture
GeneGroup
Carrier
Non-Carrier

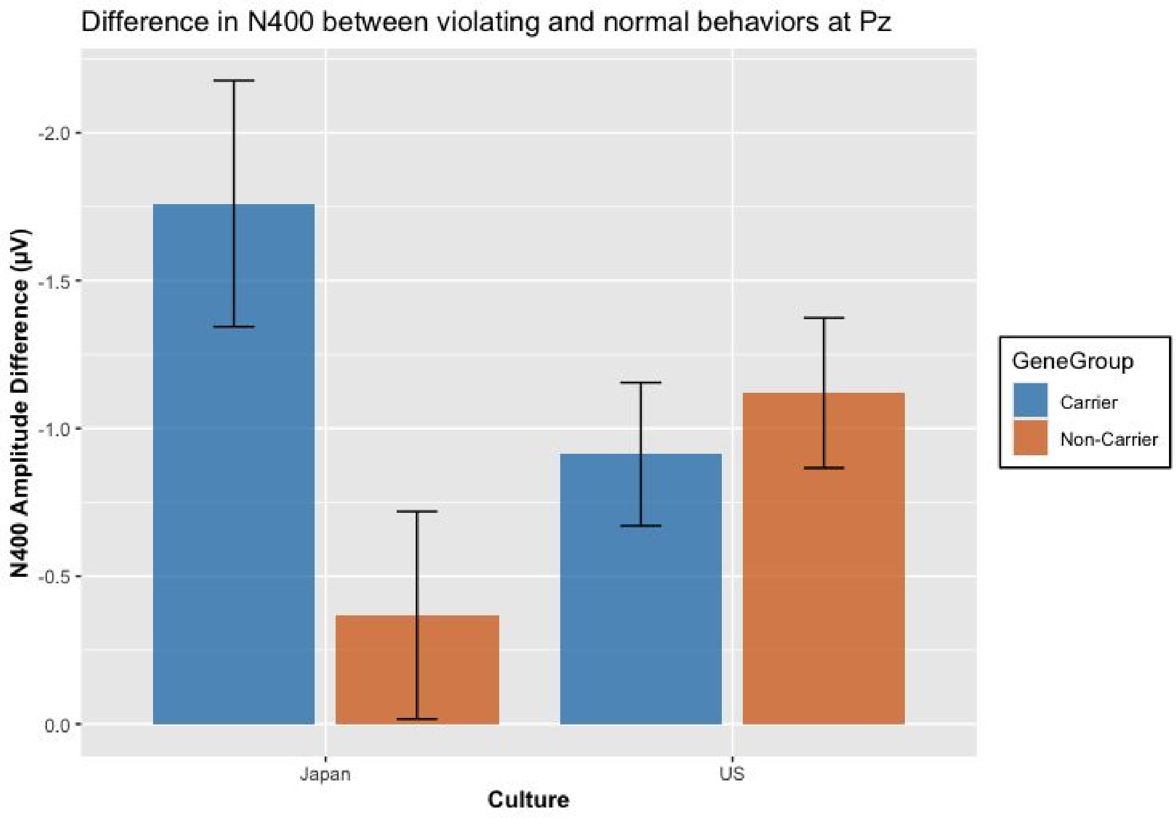
Difference in N400 between violating and normal behaviors at Pz
N400 Amplitude Difference (µV)
-2.0
-1.5
-1.0
-0.5
0.0
Japan
US
Culture
GeneGroup
Carrier
Non-Carrier

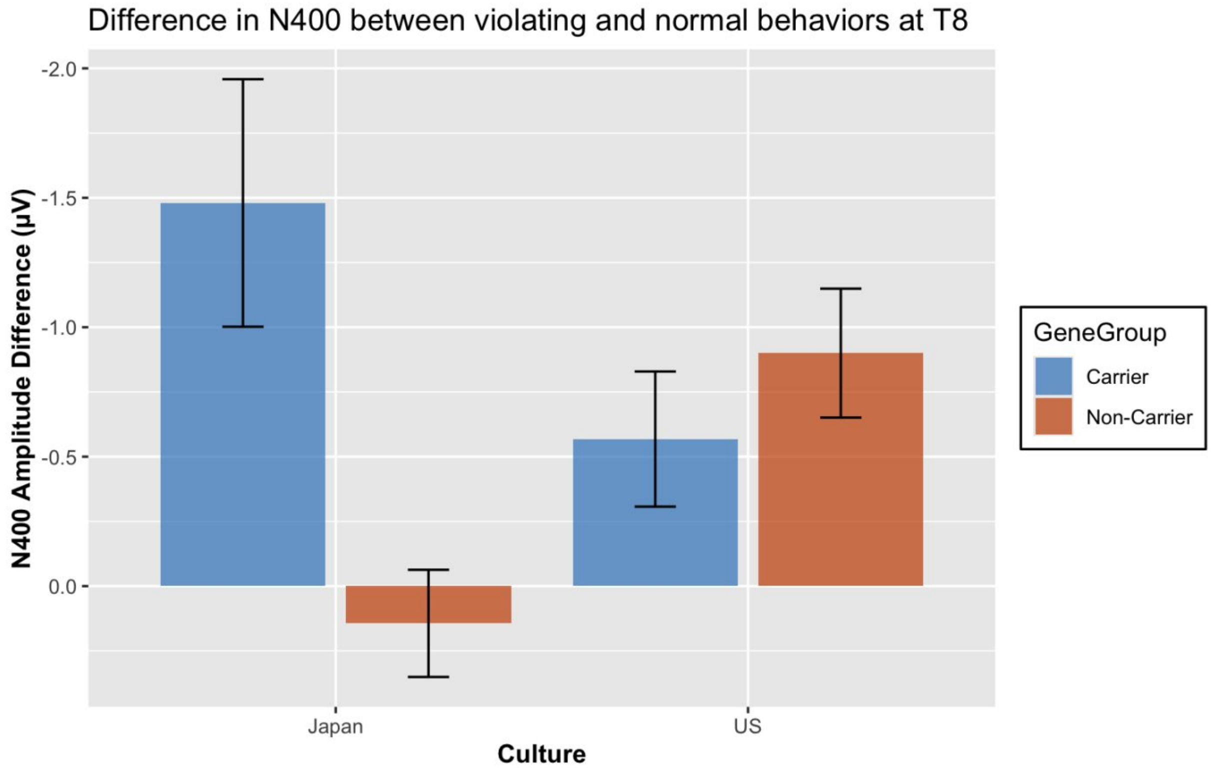
Difference in N400 between violating and normal behaviors at T8
N400 Amplitude Difference (µV)
-2.0
-1.5
-1.0
-0.5
0.0
Japan
US
Culture
GeneGroup
Carrier
Non-Carrier
